# Phagocytotic impairment of tissue-resident alveolar macrophages by diesel particulates drives pulmonary surfactant accumulation

**DOI:** 10.1007/s44307-026-00113-y

**Published:** 2026-05-19

**Authors:** Ruiqi Chen, Zhenkun Zhou, Jing Wang, Yilin Zhang, Peibo Li, Hao Wu

**Affiliations:** 1 School of Life Sciences, Guangdong Engineering & Technology Research Center for Quality and Efficacy Reevaluation of Post-Market Traditional Chinese Medicine, Sun Yat-sen University, Guangzhou, 510275 P.R. China; 2 Guangdong Provincial Key Laboratory of Plant Stress Biology, State Key Laboratory of Biocontrol, Sun Yat-sen University, Guangzhou, 510275 P.R. China; 3 Guangdong Provincial Biotechnology Research Institute (Guangdong Provincial Laboratory Animals Monitoring Center), Guangzhou, 510530 P.R. China; 4 Guangdong Provincial Key Laboratory of Pharmaceutical Functional Genes, School of Life Sciences, Sun Yat-sen University, Guangzhou, 510275 P.R. China

**Keywords:** Diesel particulate matter, Alveolar macrophages, Chemotaxis, Phagocytosis, Pulmonary surfactant

## Abstract

Airborne particulate matter, particularly diesel particulate matter (DPM), is a major component of air pollution and poses significant risks to lung health. Tissue-resident alveolar macrophages (TR-AMs), which dominate the immune landscape of the alveoli, are essential for immune surveillance and pulmonary surfactant homeostasis. While previous studies have reported detrimental effects of particulate matter on macrophages, little effort has been made to its impact on the key physiological functions—chemotaxis and phagocytosis—of TR-AMs, the most predominant macrophage lineage in the lung. We found that DPM exposure markedly altered gene expression profiling in murine TR-AM cell line MH-S, notably downregulating genes related to cytoskeleton actin dynamics, chemotaxis, and bacterial recognition. Both MH-S cells and primary TR-AMs displayed impaired migration and phagocytosis of *Escherichia coli* and *Staphylococcus aureus*, associated with reduced filamentous actin (F-actin) polymerization and filopodia formation. Cytochalasin D treatment further confirmed the role of actin remodeling in bacterial engulfment. In vivo*,* DPM exposure reduced the proportion of highly phagocytic TR-AMs, accompanied by inefficient clearance of pulmonary surfactant, leading to accumulation of Periodic acid-Schiff-positive granules, elevated surfactant protein D, and excessive lipids in both alveoli and bronchoalveolar lavage fluid (BALF). Further compositional analysis of BALF revealed excessive accumulation of phosphatidylcholine, ceramide, and cholesterol in DPM-exposed mice. In conclusion, DPM disrupts F-actin–dependent migration and phagocytosis in TR-AMs, undermining alveolar immune surveillance and surfactant homeostasis. These results highlight a key mechanism by which air pollution compromises lung defense, offering insight into particulate matter–related respiratory disease pathogenesis.

**Supplementary Information:**

The online version contains supplementary material available at 10.1007/s44307-026-00113-y.

## Introduction

With rapid industrialization and urbanization, elevated levels of airborne particulate pollution have emerged as a pressing global public health concern (Cohen et al. 2017). Airborne particulate matter is a complex mixture of tiny solid particles and liquid droplets, containing acids, organic chemicals, metals, and dust or soil particles (United States Environmental Protection Agency 2025). In urban areas, diesel particulate matter (DPM) is a major contributor to ambient particulate pollution (Ristovski et al. 2012). It is primarily emitted from diesel engines and transportation. In 2013, the International Agency for Research on Cancer classified diesel exhaust as “carcinogenic to humans” (Group 1). Despite its serious health-threatening risks, the detrimental effects of DPM remain largely underestimated. This is probably due to an inconsistent correlation between reported subjective symptoms and objective clinical signs. Although DPM exposure has been shown to impair various cell types and organs, affected individuals often experience nonspecific symptoms or merely mild discomfort. Consequently, this leads to underdiagnosis and inadequate medical interventions.

Approximately 50%–90% of DPM consists of ultrafine particles with aerodynamic diameters less than 0.1 μm, enabling deep penetration into the respiratory alveoli (Durán et al. 2004). Consequently, the alveolar spaces and the lung’s innate immune system are not only critical anatomical barriers but also primary targets of DPM-induced injury. As the most abundant immune cells in the lungs under physiological states, lung macrophages are crucial for maintaining tissue homeostasis and functional integrity (Cai et al. 2014). Based on their distinct developmental origins, anatomical locations, and functions, lung macrophages can be broadly classified into interstitial macrophages (IMs), tissue-resident alveolar macrophages (TR-AMs), and recruited macrophages (Byrne et al. 2015). IMs originate from the yolk sac. They sustain their population by either self-replenishing or through gradual replacement by monocytes. These cells primarily reside within the bronchial interstitium alongside other leukocytes, where they participate in neuroimmune communication, leukocyte recruitment, and immune modulation (Fu et al. 2022; Gibbings et al. 2017; Ural et al. 2020). Unlike IMs, TR-AMs originate from fetal liver monocytes. They maintain a long-lived, self-sustaining population within the alveolar lumen. Under steady-state, they require minimal replenishment from circulating monocytes (Woo et al. 2021). Physiologically, TR-AMs dominate the macrophage landscape in both alveoli and the lung, outnumbering IMs by approximately 8-fold (Gibbings et al. 2017). As primary sentinels and effector cells, TR-AMs play critical roles in routine immune surveillance, surfactant turnover, and the coordination of lung immune responses, effectively safeguarding pulmonary integrity (Malainou et al. 2023; Wu et al. 2023). Upon exposure to pathogens or pulmonary injury, circulating monocyte-derived macrophages (MDMs) are recruited to the lung macrophage pool, contributing to inflammation and tissue repair (Li et al. 2022; Zhang et al. 2025). However, TR-AMs are uniquely shaped by their alveolar microenvironment. Unlike steady-state IMs and primed MDMs, TR-AMs possess distinct features such as increased proliferative capacity, enhanced fatty acid metabolism, and superior phagocytotic abilities (Hou et al. 2023).

DPM exposure has been reported to be associated with an increased risk of respiratory diseases, such as pulmonary fibrosis (Jeong et al. 2021a), asthma (Nordenhäll et al. 2001; Zhao et al. 2024), and chronic obstructive pulmonary disease (COPD) (Fang et al. 2023, 2024). While most studies have focused on the effects of DPM on lung epithelial cells (Lee and Kang 2022; Reynolds et al. 2011; Shi et al. 2019; Smyth et al. 2020), relatively few have investigated its impact on lung macrophages. Existing studies mainly report excessive secretion of pro-inflammatory mediators (Ishihara et al. 2019; Pradhan et al. 2023). However, most of these investigations rely on monocyte-derived macrophages, such as the U937 and THP-1 cell lines. These models primarily represent the characteristics of IMs and recruited MDMs, rather than TR-AMs. TR-AMs are indispensable for maintaining alveolar homeostasis through bacterial phagocytosis, surfactant clearance, and efferocytosis. In contrast, how DPM affects this specific, first-line macrophage subtype remains poorly understood.

Therefore, it is of great significance to elucidate how exposure to environmental particles disrupts their physiological roles, compromises their phagocytic capacity, and disturbs lung homeostasis, ultimately driving the pathological decline of pulmonary integrity. In this study, we aimed to fill this critical gap by investigating the effects of DPM exposure on the vital physiological functions of TR-AMs—chemotactic mobility and phagocytosis—as well as the resulting pulmonary dysfunctions and potential underlying mechanisms.

## Materials and methods

### Cell culture and DPM exposure

The murine alveolar macrophages (AMs) cell line MH-S (Procell, Wuhan, China) and murine lung epithelial cell line TC-1 (Otwo, Shenzhen, China) were cultured in RPMI Medium 1640 (Gibco, Carlsbad, CA, USA) containing 10% fetal bovine serum (Cellcook, Guangzhou, China), 100 units/mL Penicillin and 100 μg/mL Streptomycin (Gibco, Carlsbad, CA, USA) at 37℃ in a humidified atmosphere of 5% CO_2_. For DPM stimulation, MH-S cells were treated with DPM (NIST, Gaithersburg, MD, USA) suspension for 24 h. Equivalent volume of culture medium was applied as vehicle.

### Cell viability

To evaluate cell viability, MH-S cells were stimulated with the indicated concentrations of DPM for 24 h (Fig. S1a and b). Following DPM stimulation, cells were stained with Calcein-AM/propidium iodide (PI) (Beyotine, Shanghai, China) for 30 min in the dark. Viable cells were identified as Calcein-AM^+^ PI^−^ cells under a confocal laser scanning microscope (SP8X, Leica, Wetzlar, Germany).

### Chemotaxis assay

To evaluate the chemotactic mobility of AMs, the co-culture trans-migration assay was applied. To prepare conditioned medium, MH-S cells were treated with vehicle or DPM (200 μg/mL) for 24 h, followed by co-incubation with pHrodoRed-conjugated *Staphylococcus aureus* (*S. aureus)* (0.1 mg/mL) for additional 2 h. Supernatant was collected, centrifuged (1, 500 × *g*, 5 min, 4℃), and filtered through 0.22 μm membrane. To perform chemotaxis assay, 2 × 10^5^ of vehicle or DPM treated cells were seeded onto the upper transwell insert (24 well PET transwell, 8 μm pore, Falcon, Corning, NY, USA) with normal culture medium until confluent. The basolateral normal culture medium was replaced by 400 μL of control medium or DPM conditioned medium, and allowed for trans-migration for 4 h. After 4 h incubation, the culture inserts were rinsed with PBS, fixed with 4% paraformaldehyde, and stained with 0.5% crystal violet (Aladdin, Shanghai, China). Non-migrated apical cells were scraped off, and images of the migrated cells were captured using inverted microscope (ECLIPSE Ts2, Nikon Instruments Inc., Tokyo, Japan) and analysed by ImageJ software, version 1.53 (National Institutes of Health, Bethesda, MD, USA).

### Chemokine array assay

Profiling of pro-inflammatory chemokines in MH-S cells was achieved by using Proteome Profiler Mouse ChemokineArray kit (R&D Systems, Minneapolis, MN, USA). Briefly, to ensure the representativeness of the data, MH-S cells were seeded in T75 flasks. Vehicle or DPM treated MH-S cells were co-incubated with pHrodoRed *Escherichia coli* (*E. coli*) or *S. aureus* for 2 h. Then culture supernatant was collected, centrifuged (1, 500 × *g*, 5 min, 4℃), filtered through 0.22 μm. 0.5 mL of vehicle control medium or DPM conditioned medium was applied to Chemokine Array assay according to manufacturer’s instruction. The relative intensities of each dot were quantified by ImageJ software.

### RNA sequencing

Total RNA of vehicle or DPM treated MH-S cells were collected using Trizol reagent (Ambion, Calsbad, CA, USA). cDNA was generated by the reverse-transcription of the cleaved RNA fragments using SuperScript™ II Reverse Transcriptase (Invitrogen, cat. 1896649, USA). The library was constructed and paired-end sequencing (PE150 mode) was performed on an Illumina Novaseq™ 6000 (LC-Bio Technology CO., Ltd., Hangzhou, China). Data were mapped to the reference genome, genes with |log 2 ratio|≥ 1 and *p*-value < 0.05 were selected for data analysis. Significant differentially expressed genes (DEGs) were identified by referring to the criteria of *p* value < 0.05 and |log 2fold change|> 1.

### Cholesterol uptake assay

To evaluate the impact of DPM on the cholesterol uptake capacity of MH-S cells, MH-S cells were pretreated with vehicle or 200 μg/mL DPM for 24 h, followed by 2 h co-incubation with 5 μM BODIPY-Cholesterol (MCE, Monmouth Junction, NJ, USA). Then, MH-S cells were lysed with 0.1 M NaOH overnight. The cell lysates and the residual cholesterol in culture supernatants were centrifuged (13, 000 × *g*, 30 min, 4℃) and then the supernatants were measured using fluorescence microplate reader (Spark, Tecan, Männedorf, Switzerland). Fluorescence intensity was quantified as relative fluorescence unit (RFU).

### Preparation and treatment of conditioned media

To distinguish the direct impact of DPM on MH-S from the indirect effects mediated by lung epithelial cells, an in vitro cross-treatment model was established. Briefly, murine lung epithelial TC-1 cells were treated with vehicle or 200 μg/mL DPM for 6 h. The culture supernatants were collected, centrifuged (1, 500 × g, 10 min, 4℃), and filtered through 0.22 μm membrane to prepare TC-1 Ctrl or DPM conditioned medium. MH-S cells were incubated with this conditioned medium for 24 h with or without direct DPM exposure (200 μg/mL), followed by the evaluation of their phagocytic capacity.

### Animal model

Male BALB/c mice (18—22 g, 6—8-week-old) were purchased from the Experimental Animal Center of Sun Yat-sen University (Guangzhou, China) and were housed in specific pathogen free environment with 12:12 h light–dark cycle, temperature at 21°C—23℃ and humidity of 45%−65%. All animal experimental protocols have been implemented in compliance with the regulations of the Animal Care and Use Committee of Sun Yat-Sen University (Ethics approval No. SYSU-IACUC-2021-000259, Guangzhou, China). Every effort was made to minimize both the employed animal numbers and the potential discomforts of the animals throughout the study.

For DPM stimulation, mice were anesthetized using isoflurane inhalation. 50 μL DPM solution (15 mg/kg) was administrated via intratracheal instillation, twice, on Day 1 and Day 3, respectively. The 15 mg/kg dose was chosen based on established acute mouse exposure protocols using comparable dose particulate administration that reliably induce pulmonary immunomodulation and injury (Jeong et al. 2021b; Yang et al. 2003a). 50 μL sterile saline was delivered as vehicle for control (Ctrl) mice. 24 h after the second DPM administration, mice were sacrificed for sampling.

To evaluate the in vivo phagocytosis of TR-AMs, on Day 4, 50 μL of pHrodoRed-AF488 *E. coli* (0.3 mg/mL) or pHrodoRed-AF488 *S. aureus* (0.3 mg/mL) (Invitrogen, Carlsbad, CA, USA) was intratracheally administrated into the mouse. After 3 h, the bronchoalveolar lavage fluid (BALF) was collected for flow cytometry analysis. To further investigate the long-term effects of DPM exposure, at 1 day, 2 weeks, 4 weeks, and 8 weeks post the final instillation of DPM, the in vivo phagocytosis of TR-AMs against *S. aureus* was evaluated using the same method.

### Collection of mice BALF

24 h post the second DPM delivery, pulmonary epithelial lining fluid was collected by bronchoalveolar lavage. Briefly, BALF was collected by 1 instillation and recovery of 1 mL of cold saline into the trachea. The BALF was centrifuged (1,000 × *g*, 5 min, 4℃), the supernatant was stored at −80℃ till use.

### Purification of AMs from BALF

The BALF was centrifuged (1,000 × g, 5 min, 4℃). The cell pellets were incubated with red blood cell lysis (10 min, on ice), rinsed with cold PBS, and centrifuged (500 × *g*, 5 min, 4℃). Total cell counts were determined by a hemocytometer, and seeded for adherent for 2 h at 37℃ incubators. The non-adherent cells were wash off, and the purity of adherent AMs were examined by SiglecF staining.

### Flow cytometry

After DPM stimulation, MH-S cells were stained with monoclonal antibodies (Biolegend, San Diego, CA, USA) anti-ms-CD11a-PE (121/7), anti-ms-CD11b-AlexaFluor700 (M1/70), anti-ms-CD45.2-PB (104). Stained cells were analyzed by flow cytometry (CytoFlex, Beckman Coulter, Brea, CA, USA). Mean fluorescence intensity (MFI) was quantified using FlowJo software, version 10.6.2 (FlowJo LLC, Ashland, OR, USA).

To evaluate phagocytosis in vitro, cells were incubated with AF488-conjugated pHrodoRed *E. coli* (0.2 mg/mL) or *S. aureus* (0.25 mg/mL) bioparticles. After incubation, particle engulf cells were analyzed by flow cytometry (MoFlo Astrios EQs, Beckman Coulter, Brea, CA, USA). To verify the involvement of actin polymerization, MH-S cells were pretreated with 2.5 μM cytochalasin D for 1 h prior to either naïve staining or pHrodoRed bioparticles incubation.

To measure the phagocytotic AMs in vivo, purified AMs were isolated from the BALF of vehicle or DPM treated mice after AF488-conjugated pHrodoRed bioparticles administrated for 3 h. After Fc block, AMs were surface stained with anti-ms-CD45.2-PB (104), anti-ms-SiglecF-APC (S17007L), and analyzed by flow cytometry (MoFlo Astrios EQs, Beckman Coulter, Brea, CA, USA). The MFI of pHrodoRed and AF488 were quantified in CD45.2^high^SiglecF^+^TR-AMs gating.

To verify the purity of TR-AMs in our gating strategy, cells were isolated from the BALF of vehicle or DPM treated mice. They were surface stained with anti-ms-CD45.2- PB (104), anti-ms-SiglecF-APC (S17007L), anti-ms-CD11c-BV650 (N418) and anti-ms-CD11b-PE (M1/70) after Fc block. Cells were analyzed using flow cytometry (CytoFlex LX, Beckman Coulter, Brea, CA, USA).

### Immunofluorescence

For evaluation of phagocytosis in vitro, cells on petri dish or µ-Slide 8-well coverslips (ibidi, Martinsried, Germany) were fixed with 4% paraformaldehyde for 30 min and permeabilized with 0.1% triton X-100. Cells were stained with AF488 phalloidin (Invitrogen, Carlsbad, CA, USA) for 10 min in the dark, and counterstained with Hoechst 33,342. To evaluate phagocytosis in vivo, TR-AMs were purified from BALF of vehicle or DPM treated mice after AF488-conjugated pHrodoRed bioparticles administrated for 3 h, after red blood cell lysis and Fc block, fixed cells were stained with anti-ms-SiglecF-APC (Clone S17007L, Biolegend, San Diego, CA, USA), AF488 phalloidin, counterstained with Hoechst, and mounted with antifade medium. Immunofluorescent images were visualized by a confocal laser scanning microscope (SP8X, Leica, Wetzlar, Germany). The expression of phalloidin as well as the intracellular intensity of pHrodoRed *S. aureus* were quantified by ImageJ software.

### Reverse Transcription Quantitative PCR (RT-qPCR)

Total RNA was isolated using EZ-press RNA Purification Kit (EZBioscience, Suzhou, China) or RNAprep Pure Micro KitRNA (TIANGEN, Beijing, China) according to the manufacturer’s instruction. Concentration and quality (OD260/OD280) were determined using NanoDrop 2000 C spectrophotometer (Thermo Fisher Scientific, Waltham, MA, USA). 1 μg of RNA was reversely transcribed into cDNA using the FastKing gDNA Dispelling RT SuperMix (TIANGEN, Beijing, China). For each sample, PCR was performed in triplicates with SuperReal PreMix Plus (SYBR Green) (TIANGEN, Beijing, China) and the RT-qPCR platform (LC480, Roche, Basel, Switzerland). Relative abundance of each target gene was normalized to that of the housekeeping gene β-actin. Primers were listed in Supplementary Table 1.

### ELISA and colorimetric assay

Levels of surfactant protein A (SP-A) (Cloud-Clone Corp., Wuhan, China), surfactant protein D (SP-D) (Cloud-Clone Corp., Wuhan, China) in BALF was quantified by enzyme-linked immunosorbent kits. Measurement of total protein contents (BCA method, Beyotime Biotech Inc., Shanghai, China), Lactate dehydrogenase (LDH) activity (Solarbio Life Sciences, Beijing, China), and free cholesterol levels (Beyotime Biotech Inc., Shanghai, China) in BALF were achieved by colorimetric kits.

### Histology

On Day 4, mice lung was perfused with 4% paraformaldehyde, collected, and post-fixed in 4% paraformaldehyde for 24 h, dehydrated with gradient ethanol, and cut into 5 μm paraffin slides. For Periodic acid-Schiff (PAS) staining, after deparaffinization, slides were stained with 0.5% Periodic acid for 5 min, rinsed in distilled water, followed by staining in Schiff’s reagent for 15 min, rinsed in running tap water, and counterstained with hematoxylin. The full scan lung histology was captured (Axioscan7 digital slide scanner, Carl Zeiss, Germany) and evaluated by two independent observers who were blinded to experiment settings.

### Extraction of lipids from BALF

BALF lipids were extracted by liquid–liquid extraction method. Briefly, 60 μL BALF were mixed with 340 μL H_2_O, 960 μL of methyl tert-butyl ether (methyl tert-butyl ether/MeOH, 5:1, *v/v*), and 2 μL of Internal control (SPLASH LIPIDOMIX internal standards, Avanti Polar Lipids, Cat# 330709, Alabaster, AL, USA). Samples were vortexed vigorously for 60 s, followed by sonication (10 min, 4 °C water bath) and centrifugation (1,000 × *g*, 15 min, 4 °C). 500 μL supernatant were transferred into new tube, and 500 μL methyl tert-butyl ether was added to the remaining solution for re-extraction. A total of three times methyl tert-butyl ether extraction was applied. 1.5 mL of combined supernatant were evaporated to dryness (4 °C, vacuum concentrator), stored at −80 °C till use.

Prior analysis, dried pellets were re-dissolved in 160 μL of methylene chloride/MeOH (1:1, v/v), vortexed to dissolve and centrifuged (15,000 × *g*, 30 min, 4 °C). For target lipidomics analysis, 8 μL of supernatant was injected into ultra-performance liquid chromatography tandem triple quadrupole mass spectrometry (UPLC- Triple Quad -MS/MS). Quality control samples were prepared by pooling 30 μL of each sample, and were applied at an interval of every 7 to 8 samples for batch correction and data normalization.

### Targeted lipidomics analysis

Full lipidome was analyzed by multiple reaction monitoring using a connected system of AQUITY UPLC (Waters Corp., Milford, MA, USA) -hybrid triple quadrupole mass spectrometer (Triple Quad™ 5500, AB Sciex, Foster City, CA, USA) with electrospray ionization source. We adapted a protocol by modifying the published method described by Xuan et al. (2018). For each specific lipid class, five separate injections were set up per sample, and multiple reaction monitoring transitions were set up for analysis of various polar lipids. Separation of lipids was achieved on a Waters BHT C8 1.8-μm column (internal diameter 2.1 × 100 mm) with column temperature at 55◦C. The mobile phase system consists of acetonitrile: H_2_O (60:40 v/v) (A) and Isopropanol: acetonitrile (90:10 v/v) (B), both containing 10 mM ammonium acetate at a flow rate of 260 μL per min for 21 min. The gradient elution was set as follows: 32%—85% B (1.5—15.5 min), 85%—97% B (15.5—15.6 min), maintained at 97% B (15.6—18.0 min), and back to the original 32% B to equilibrate the system (18.1—21 min). Experimental parameters for mass spectrometry: ion source gas 1 and gas 2 were both 45 psi, curtain gas was 35 psi, ion source temperature was 600 °C, ion spray voltage floating was 5,500 V in positive mode while −4,500 V in negative mode, and collision gas was medium.

Data initialization checks were performed using the Explorer module in Sciex OS software, version 1.4.1.20719 (AB Sciex, Foster City, CA, USA), and peak table extraction and processing were conducted using the Analyst module. Processing and statistical analyses for lipidomics data was performed using MetaboAnalyst 6.0 (https://www.metaboanalyst.ca/). The intensities of different lipid classes were normalized by internal standards or the total sum of peak intensities within each class. Then, the data underwent Log_10_ transformation and auto-scaling. Significantly differentially abundant lipid species were identified based on the criteria of a False Discovery Rate < 0.05 and |log 2fold change|> 1.

### Statistical analysis

Statistical analysis was conducted by SPSS software, version 22.0 (IBM Corp., Armonk, NY, USA) and GraphPad Prism software, version 8.0.1 (GraphPad Software Inc., La Jolla, CA, USA). Data were presented as mean ± SEM. The statistical analysis was assessed using unpaired t-test or one-way ANOVA followed by Bonferroni post-hoc test, as indicated. A value of *p* < 0.05 was considered statistically significant.

## Results

### DPM induces widespread gene expression alterations in murine AM MH-S cells

**Fig. 1:**
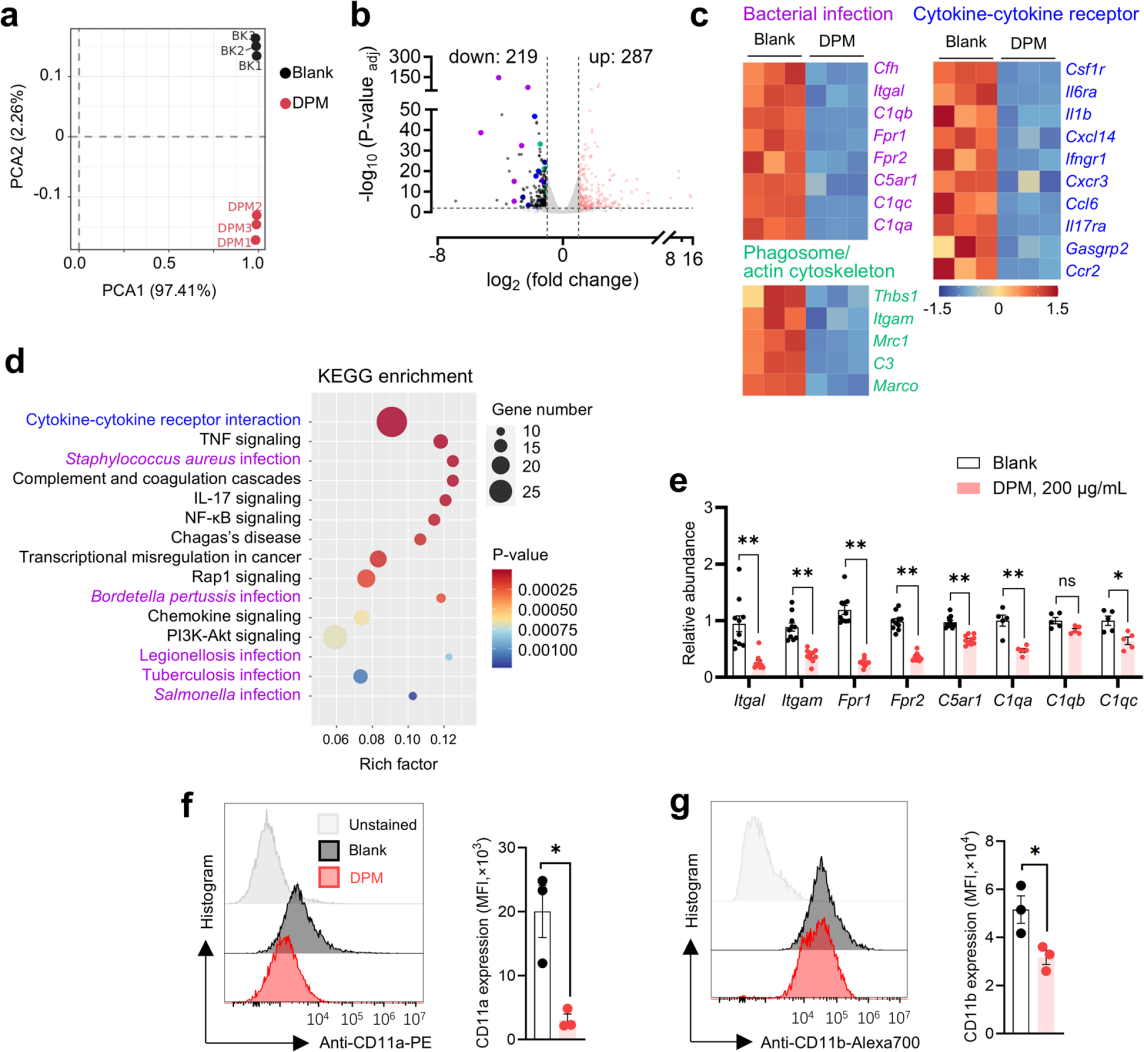
DPM caused broad gene expression alterations in MH-S cells. **a** PCA plot of gene expression via RNA sequencing after 24 h vehicle or DPM stimulation (*n* = 3). **b** Volcano plot of DEGs. A total of 219 down-regulated and 287 up-regulated genes were identified. **c** Heatmap of DEGs functional related to bacterial infection (magenta), cytokine-cytokine receptor interactions (blue), phagosome and actin cytoskeleton (green). **d** Kyoto Encyclopedia of Genes and Genomes enrichment of DEGs. **e** Abundance of differentially expressed genes by RT-qPCR (*p < 0.05, **p < 0.01, *n* = 5). **f**—**g** Levels of CD11a (**f**) and CD11b (**g**) of vehicle or DPM treated MH-S cells were measured by flow cytometry. Histograms were presented and MFI were quantified (*p < 0.05, *n* = 3)

Unlike other lung macrophages, TR-AMs exhibit distinct phenotypes and transcriptional signatures shaped by lung microenvironment (Gautier et al. 2012). To investigate how DPM exposure alters AM function, we performed RNA sequencing analysis. The murine AM cell line MH-S was exposed to vehicle or DPM for 24 h before sequencing. Principal component analysis (PCA) revealed substantial gene alterations in DPM-treated cells (Fig. 1a), with 287 genes upregulated and 219 downregulated (Fig. 1b). Pathway enrichment analysis highlighted disruptions in phagosome/actin cytoskeleton regulation (*Thbs1*, *Itgam*, *Mrc1*, *C3*, *Marco*), bacterial recognition (*Cfh*, *Itgal*, *Fpr1*, *Fpr2*, *C5ar1*, *C1qa*, *C1qb*, *C1qc*), and cytokine-cytokine receptor interactions (*Csf1r*, *Il6ra*, *Il1b*, *Ifngr1*, *Cxcr3*, *Ccl6*, *Il17ra*, *Ccr2*) (Fig. 1c and d). RT-qPCR validated that DPM significantly suppressed the expressions of *Itgal*, *Itgam*, *Fpr1*, *Fpr2*, *C5ar1*, *C1qa*, and *C1qc* (Fig. 1e). Flow cytometry further confirmed reduced surface expression of CD11a (*Itgal*) (Fig. 1f) and CD11b (*Itgam*) (Fig. 1g) following DPM exposure. Given that these integrins are essential for immune cell adhesion, migration, and bacterial uptake, these findings suggest that DPM might affect AM motility and phagocytic capacity.

### DPM suppresses chemoattractant trans-migration of MH-S cells

**Fig. 2:**
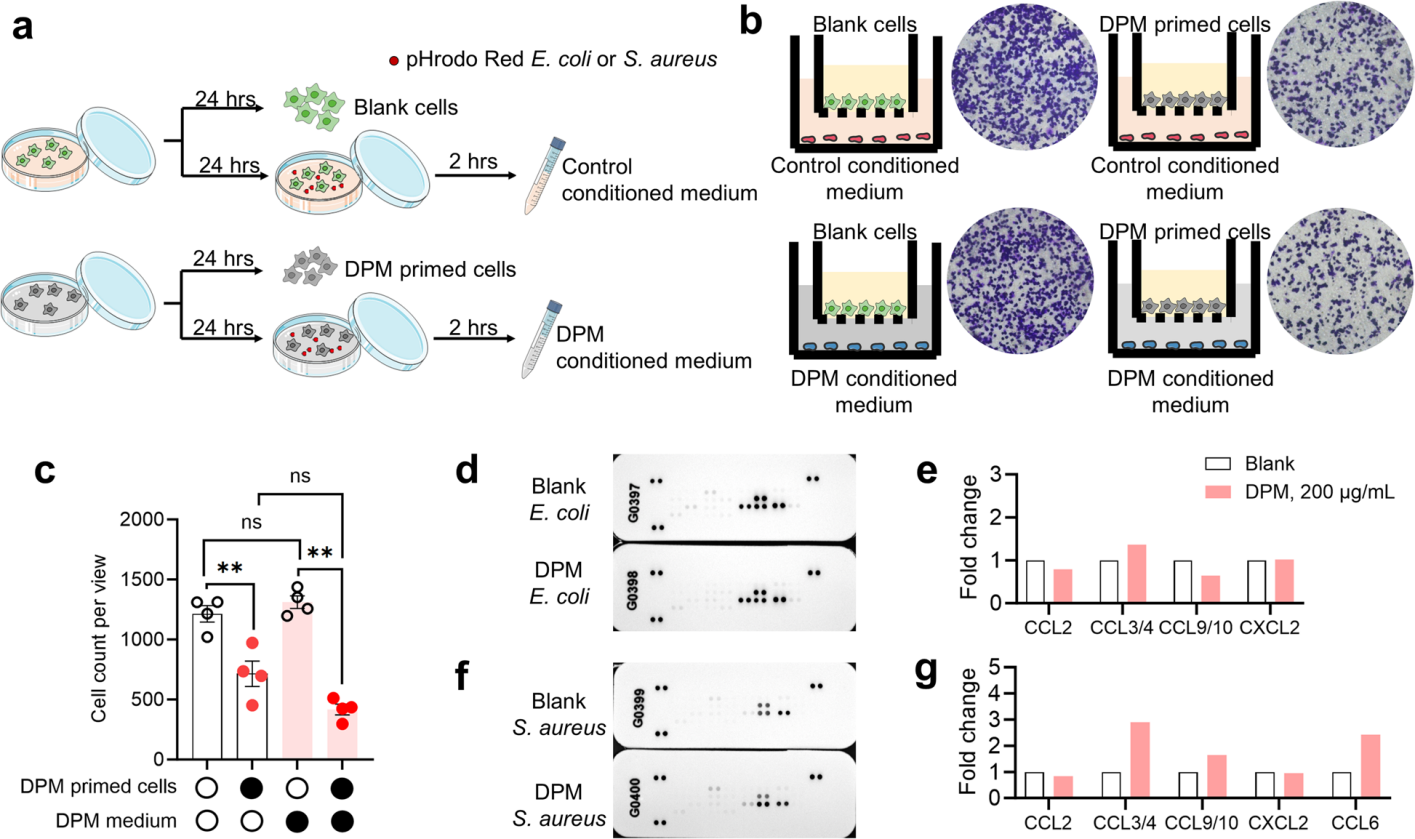
DPM compromised the chemotactic motility of AMs in vitro. **a** Schematic diagram of the experimental design, murine AM cell line MH-S was used. **b** After 4 h co-incubation, basolateral transmigrated cells were stained with crystal violet and examined. **c** Statistics of (**b**), presented as cell counts per view (**p < 0.01, *n* = 4). **d** Vehicle or DPM treated MH-S cells were co-incubated with pHrodoRed *E. coli* for 2 h. Control culture medium and DPM conditional medium were collected for chemokine array assay. **e** Quantification of dot intensity of **d** (*n* = 1), presented as normalized fold change. **f** Vehicle or DPM treated cells were co-incubated with pHrodoRed *S. aureus* for 2 h. Supernatant were collected for chemokine array assay. **g** Quantitative of **f** (*n* = 1)

Efficient alveolar immune surveillance relies on chemotaxis, which enables AMs to sense and migrate toward chemoattractants produced at localized infection sites (Kay et al. 2008). To evaluate whether DPM impairs this critical process, we performed a transwell migration assay (Fig. 2a). Conditioned medium was prepared by incubating MH-S cells with *S. aureus* bioparticles for 2 h. DPM exposure dramatically suppressed the transmigration of AMs to the dorsal side of culture inserts (Fig. 2b and c). AMs can also orchestrate host immunity by secreting chemokines that recruit other immune cells such as neutrophils and T cells. To assess whether DPM affects chemokine secretion, we conducted a migration assay using DPM primed conditioned medium. Regardless of prior exposure, there was no significant difference in cell transmigration when co-cultured with DPM-conditioned medium (Fig. 2b and c). To obtain a broad overview of MH-S chemokine secretion, we conducted a chemokine array assay. We observed comparable secretion trends for C–C motif chemokine ligands (CCL2, CCL3/4, CCL9/10 and CCL6) and C-X-C motif chemokine ligand 2 (CXCL2) in cell supernatants between groups (Fig. 2d-g). These results suggest that DPM primarily impairs AM chemotactic receptor signaling and motility rather than chemokine production. These data are primarily used for exploratory screening. Definitive conclusions regarding these chemokines require subsequent validation in isolated TR-AMs.

### DPM impairs the phagocytotic capacity of AMs

**Fig. 3:**
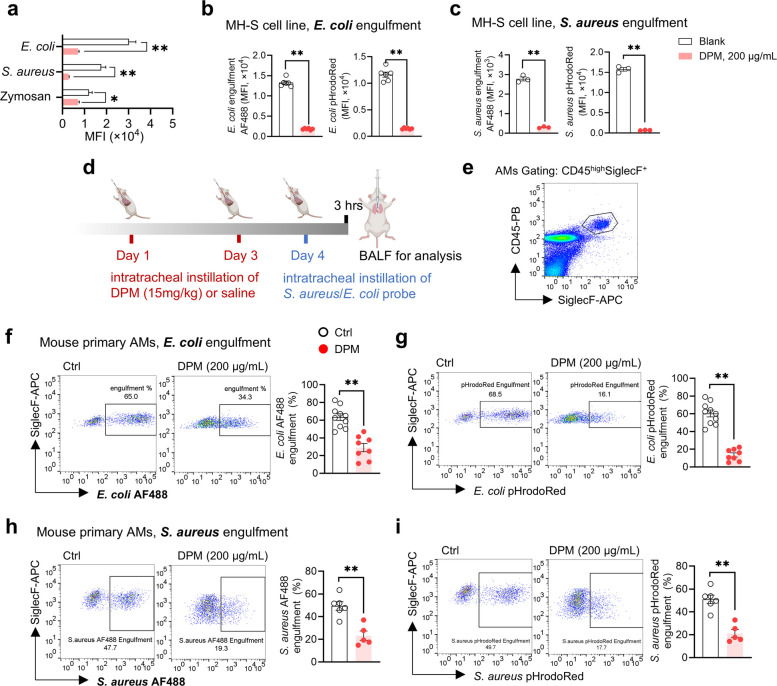
DPM dramatically compromised the phagocytotic capacity of AMs. **a** MH-S cells were pretreated with DPM or vehicle for 24 h, followed by 1 h co-incubation with pHrodoRed-conjugated bioparticles *E. coli*, *S. aureus* or Zymosan. Cellular phagocytosis was evaluated by flow cytometry, quantified as MFI. **b**—**c** MH-S cells were pretreated with DPM or vehicle for 24 h, followed by 1 h co-incubation with AF488-conjugated pHrodo Red *E. coli* (**b**) or *S. aureus* (**c**), respectively. Intracellular engulfment of bioparticles was quantified as MFI (**p < 0.01, *n* = 3—5). **d** Schematic diagram of the in vivo study design. **e** Flow cytometry gating strategy for identifying primary tissue-resident AMs (TR-AMs, CD45^high^SiglecF^+^) in BALF. **f**—**i** TR-AMs were isolated from the BALF of vehicle or DPM treated mice after AF488-conjugated pHrodoRed *E. coli* (**f**—**g**) or *S. aureus* (**h**—**i**) administrated for 3 h. Percentage of efficient phagocytic cells were presented and quantified (**p < 0.01, *n* = 5—8)

As professional phagocytes in the alveoli, AMs play a crucial role in engulfing and clearing inhaled particles, microbes, and apoptotic cells through phagocytosis—a process that enables the uptake and elimination of debris larger than 0.5 μm in diameter (Aderem 2003; Gordon 2016). Consistent with the observed downregulation of phagocytosis-related genes (Fig. 1c and d) and compromised chemotaxis, we next investigated the direct impact of DPM exposure on the phagocytic capacity of MH-S cells and primary AMs. MH-S cells were incubated with pHrodoRed-conjugated *E. coli*, *S. aureus*, or Zymosan for 1 h following 24-h stimulation with either DPM or vehicle. DPM-treated cells exhibited dramatically reduced phagocytosis of pHrodoRed-conjugated bioparticles, particularly *E. coli* and *S. aureus* (Fig. 3a). Since pHrodo fluorescence intensifies upon phagosome acidification, this assay reflects both bioparticle uptake and subsequent phagosome maturation. To specifically evaluate internalization, *E. coli* and *S. aureus*-conjugated with pHrodoRed were co-labeled with AF488 TFP ester, a pH-insensitive fluorophore. The MFIs of both pHrodoRed and AF488 were significantly reduced in DPM-exposed cells (Fig. 3b and c), indicating that DPM suppresses phagocytosis by inhibiting both particle engulfment and phagosome maturation.

To confirm these findings in vivo, we established a mouse model of DPM exposure. Mice received two intratracheal instillations of DPM, followed by instillations of pHrodoRed-AF488-conjugated *E. coli* or *S. aureus* (Fig. 3d). Primary TR-AMs were identified by CD45^high^SiglecF^+^ gating (Fig. 3e) and analyzed for bacterial phagocytosis. The strategy for identifying TR-AMs was further verified by their characteristic CD11c^high^CD11b^low^ phenotype in vehicle (Fig. S2a) and DPM-treated mice (Fig. S2b). This specific gating effectively excludes eosinophils (CD11c^−^CD11b^high^) and recruited MDMs (CD11c^low^CD11b^high^), ensuring a purified TR-AM cluster for subsequent functional analysis. Consistent with in vitro studies, TR-AMs from DPM-exposed mice exhibited a sharp reduction in their capacity to phagocytose both *E. coli* (Fig. 3f and g) and *S. aureus* (Fig. 3h and i). Furthermore, the proportion of highly phagocytic TR-AMs was substantially reduced, suggesting that DPM exposure also impaired the chemotactic recruitment of these phagocytic TR-AMs.

Since our exposure strategy followed an acute model, we sought to determine whether the observed TR-AM impairment was persistent or could gradually recover. The long-term impact of DPM was evaluated at 1 day, 2 weeks, 4 weeks, and 8 weeks after the final instillation. The phagocytic capacity of TR-AMs was significantly decreased in DPM treated mice, as evidenced by reduced cellular MFIs of both AF488-labeled *S. aureus* and pHrodo Red *S. aureus* (Fig. S3a and b). Importantly, this suppressed phagocytic capacity demonstrated a recovery trend over the 8-week observation period. Furthermore, while the intracellular bacterial burden remained consistently low, the proportion of highly phagocytic TR-AMs initially declined, and had largely recovered by 4 weeks post-exposure (Fig. S3c and d). This difference indicates that exposure to DPM leads to a sustained deficiency in phagocytic capacity that persists even after bacterial recognition and sensing mechanisms have largely normalized.

Because AMs reside in a complex alveolar microenvironment, we further investigated whether this phagocytic inhibition is driven directly by DPM or indirectly via soluble mediators secreted by other cell populations. Utilizing an in vitro conditioned medium model (Fig. S4a), we observed that incubation with DPM-conditioned medium caused a modest decrease in intracellular MFI in MH-S cells, reflecting a minor indirect effect of epithelial-derived mediators (Fig. S4b and c). In contrast, direct exposure of MH-S cells to DPM dramatically suppressed their phagocytic capacity, regardless of whether they were co-incubated with TC-1 conditioned medium. These data suggest that the primary impairment of MH-S cells is driven by direct DPM action. Nevertheless, DPM also exerts a lesser, indirect effect on AMs via paracrine signaling from other cell types.

Collectively, these findings demonstrate that direct DPM exposure profoundly impairs AM phagocytosis, severely compromising their functional integrity to clear inhaled pathogens efficiently. Notably, while this functional suppression is long-lasting, it exhibits a gradual recovery over time.

### DPM inhibits cytoskeletal remodeling-dependent phagocytosis in AMs

**Fig. 4:**
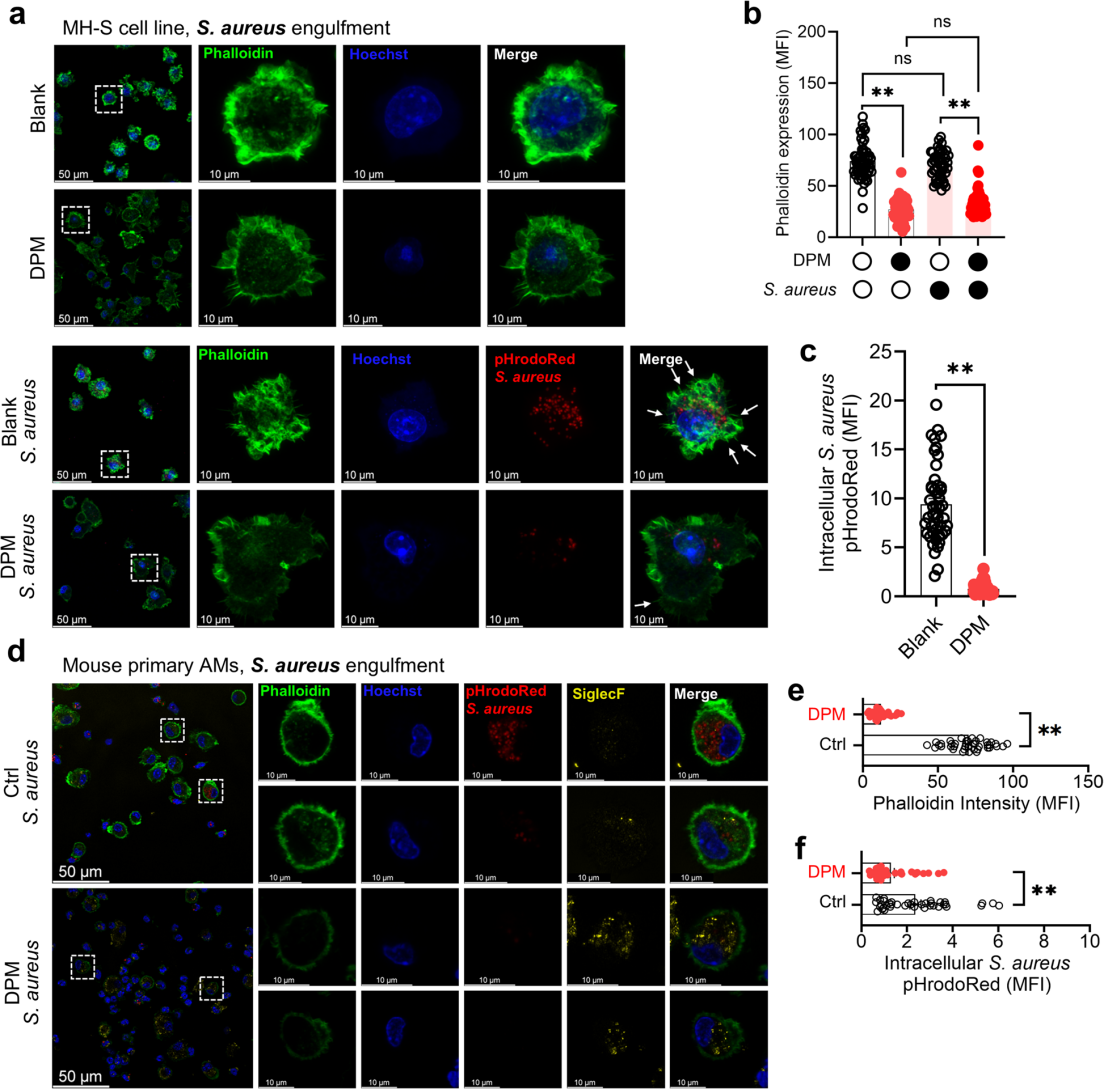
DPM inhibited AMs phagocytosis by suppressing F-actin mediated cytoskeleton rearrangement. **a**—**c** MH-S cells were treated with DPM or vehicle for 24 h, followed by 1 h co-incubation with or without pHrodoRed-conjugated *S. aureus*. **a** Representative immunofluorescence of F-actin (phalloidin, green) and engulfed *S. aureus* (red). **b** Phalloidin intensity was quantified as MFI (**p < 0.01, *n* = 43—55 cells). **c** Intracellular engulfment of *S. aureus* was quantified as MFI (**p < 0.01, *n* = 47—61 cells). **d**—**f** Mouse primary AMs were isolated from the BALF of vehicle or DPM treated mice after AF488-conjugated pHrodoRed *S. aureus* administrated for 3 h. **d** Representative immunofluorescence of F-actin (phalloidin, green), engulfed *S. aureus* (red), and SiglecF (yellow) in AMs. **e** Phalloidin intensity in AMs was quantified as MFI (**p < 0.01, *n* = 37—40 cells). **f** Intracellular engulfment of *S. aureus* was quantified as MFI (**p < 0.01, *n* = 37—40 cells)

Both migration and phagocytosis require external signal detection followed by cell body deformation driven by actin-mediated cytoskeletal remodeling (Rougerie et al. 2013). To explore whether and how DPM interferes with this process, MH-S cells were treated with DPM and then incubated with *S. aureus* bioparticles. DPM exposure significantly reduced phalloidin fluorescence intensity, in both the presence and absence of *S. aureus* bioparticle stimulation, indicating impaired filamentous actin (F-actin) polymerization (Fig. 4a and b). Moreover, the reduction in F- actin polymerization was linked to impaired phagocytosis, as DPM-treated cells showed a more than 10-fold decrease in *S. aureus* bioparticle uptake and phagosome maturation (Fig. 4c). Similarly, primary AMs from DPM-treated mice showed consistent patterns, including reduced phalloidin intensity and impaired intracellular phagocytosis of *S. aureus* bioparticles (Fig. 4d-f).

**Fig. 5:**
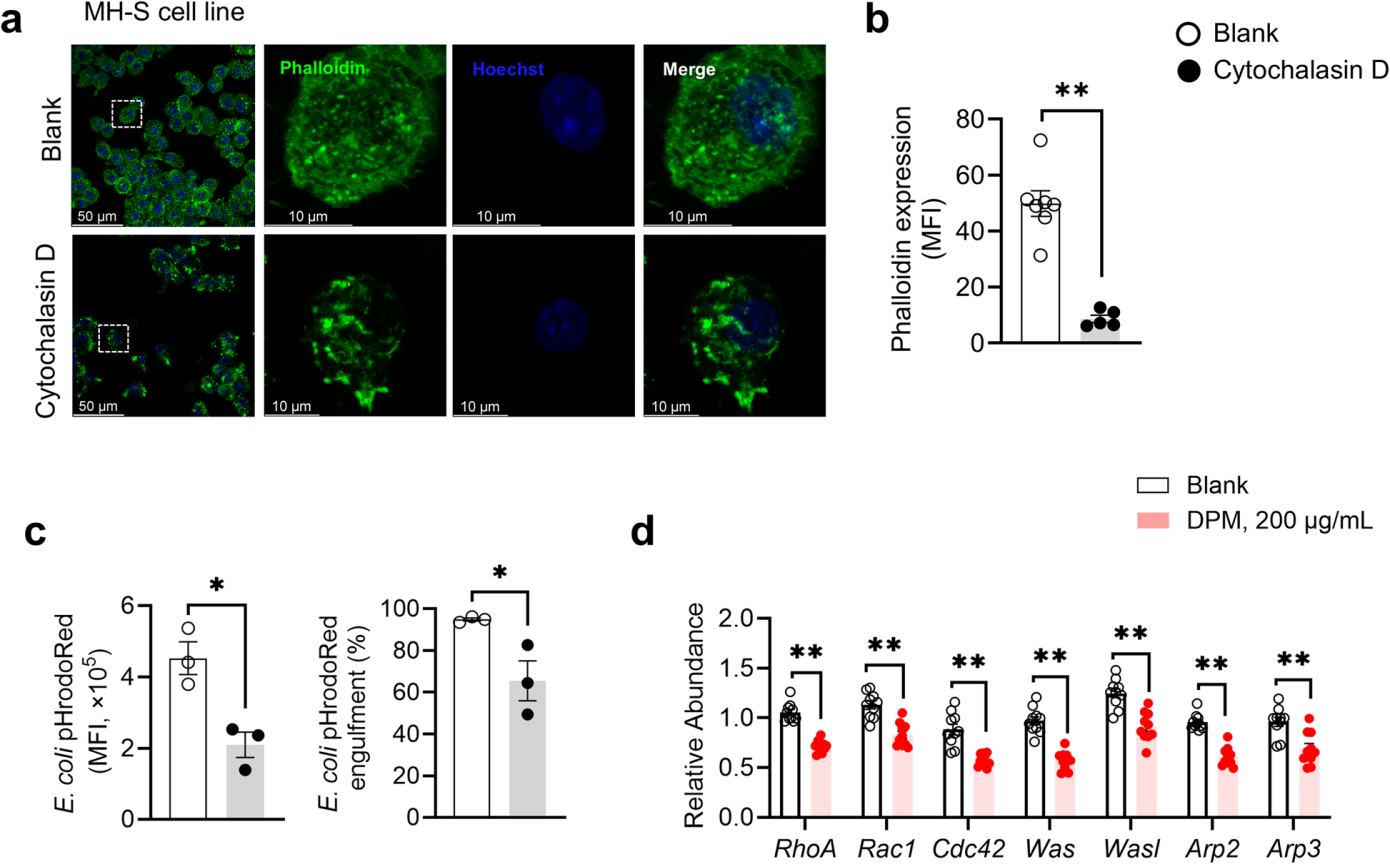
F-actin polymerization mediates AMs phagocytosis. **a** MH-S cells were treated with Cytochalasin D or vehicle for 1 h. Representative immunofluorescence of F-actin (phalloidin, green). **b** Phalloidin intensity in MH-S was quantified (**p < 0.01, *n* = 5—7). **c** MH-S cells were pretreated with Cytochalasin D or vehicle for 1 h, followed by 1 h co-incubation with pHrodoRed-conjugated bioparticles *E. coli*. Cellular phagocytosis was evaluated by flow cytometry, quantified as MFI. Percentage of efficient phagocytic cells was presented and quantified (*p < 0.05, *n* = 3). **d** Abundance of indicated genes in Rac1–N-WASP–Arp2/3 complex signaling by RT-qPCR. (*p < 0.05, **p < 0.01, *n* = 10)

To further confirm the role of actin polymerization in macrophage phagocytosis, cells were treated with cytochalasin D, a cell-permeable inhibitor that disrupts filamentous actin (Fig. 5a and b). When co-incubated with fluorescent-conjugated *E. coli* bioparticles, both the proportion of engulfing cells and the amount of engulfed bioparticles were significantly reduced following cytochalasin D treatment (Fig. 5c), indicating that F-actin-dependent cytoskeletal remodeling is essential for phagosome maturation and efficient phagocytosis. Actin nucleation and polymerization are regulated by the Arp2/3 protein complex, which is activated by the GTPases Rac1 and Cdc42 through the nucleation-promoting factors (NPFs) Wiskott-Aldrich syndrome protein (WASP), neural WASP (N-WASP), and WASP-family verprolin homologous protein (WAVE) (Rotty et al. 2013). DPM exposure significantly reduced the expression of *RhoA*, *Rac1*, *Cdc42*, *Was*, *Wasl*, *Arp2*, and *Arp3* in MH-S cells (Fig. 5d), suggesting that disruption of this signaling cascade may underlie the impaired F-actin remodeling. These findings, when taken together, suggest that DPM inhibits F-actin-mediated cytoskeletal remodeling in AMs, which contributes to their impaired phagocytic function.

### DPM-induced AM dysfunction disrupts pulmonary surfactant homeostasis

**Fig. 6:**
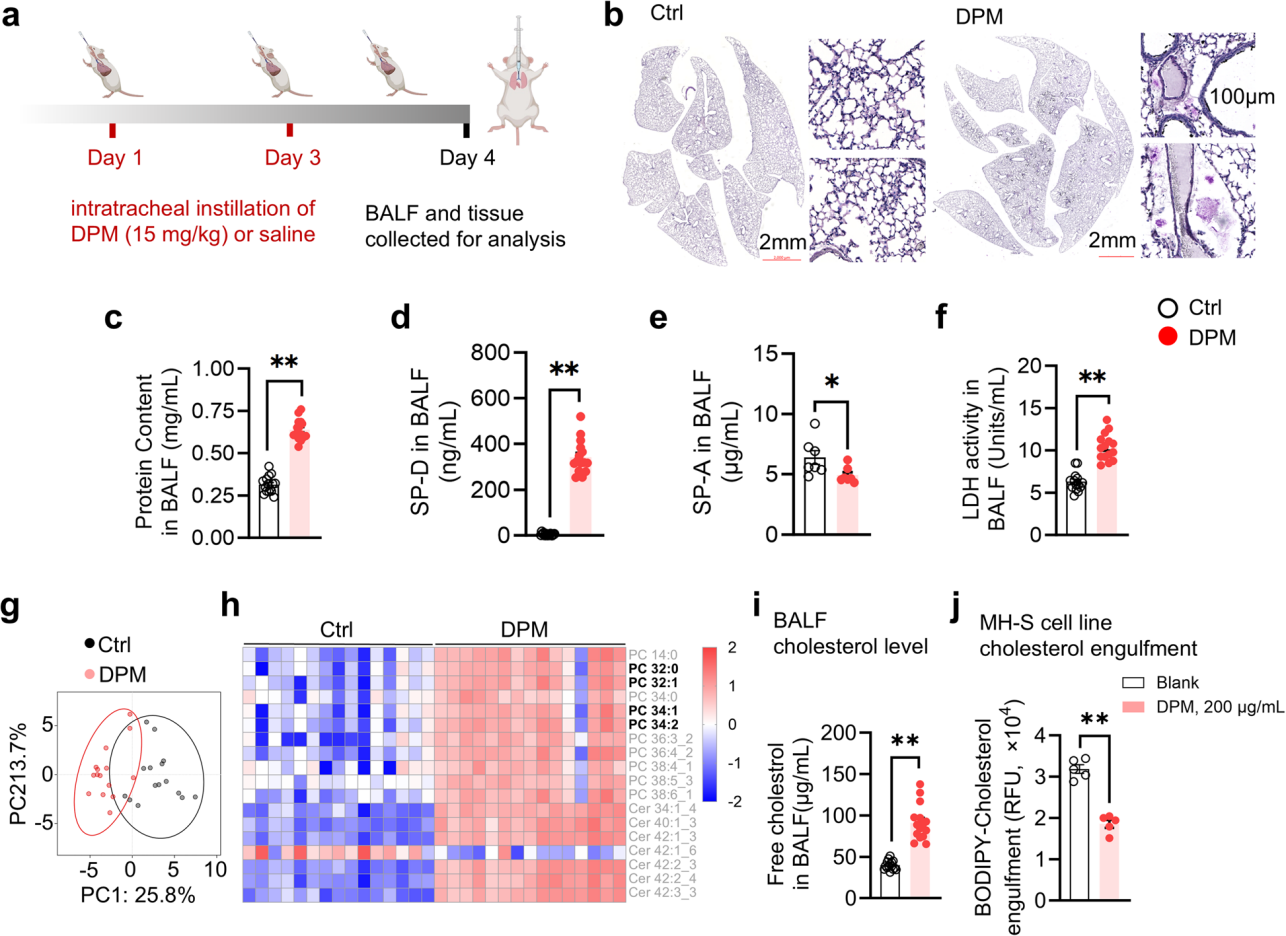
Inhaled DPM induced pulmonary surfactant accumulation in mice. **a** Schematic diagram of the in vivo study. **b** Representative lung histology of PAS staining (*n* = 5). Scale bar: 2 mm and 100 μm as indicated. **c**—**f** Contents of total protein (**c**), surfactant protein SP-D (**d**) and SP-A (**e**), and LDH activity (**f**) in BALF of vehicle or DPM treated mice (*p < 0.05, **p < 0.01, *n* = 14—15). **g**—**h** Target lipidomics of BALF from vehicle and DPM treated mice (*n* = 15). **g** PCA plot of lipids intensity. **h** Heatmap of significantly altered PCs and Cer. **i** Free cholesterol level of vehicle or DPM treated mice. **j** MH-S cells were pretreated with DPM or vehicle for 24 h, followed by 2 h co-incubation with BODIPY-Cholesterol. The engulfment of cholesterol was measured using a fluorescence microplate reader and quantified as RFU. (**p < 0.01, *n* = 5)

Pulmonary surfactant lines the alveolar surface to reduce surface tension and maintain respiratory mechanics (Whitsett et al. 2010). Because AM phagocytosis is essential for clearing excess pulmonary surfactant (Gräbner and Meerbach 1991), we hypothesized that the severe phagocytic deficits induced by DPM might disrupt pulmonary surfactant homeostasis. To investigate this, we examined DPM-exposed mice (Fig. 6a) and found irregularly shaped PAS-positive granules in the alveolar space (Fig. 6b), suggesting the abnormal accumulation of aldehyde-enriched glycans, such as surfactant glycoproteins, lipoprotein deposits, and mucins. Pulmonary surfactant consists of approximately 90% lipids and 10% proteins. This protein fraction includes surfactant proteins SP-A and SP-D—highly glycosylated collectins that stain positive with PAS (Sano and Kuroki 2005). Therefore, this histological feature is indicative of an abnormal accumulation of surfactant glycoproteins. In line with this, total protein levels in BALF were sharply elevated (Fig. 6c), along with a 120-fold increase in SP-D levels (Fig. 6d) and a slight decline in SP-A (Fig. 6e). LDH level in the BALF also suggested increased cell death after DPM instillation (Fig. 6f).

To investigate the dynamics of surfactant lipids, we performed targeted lipidomics. PCA revealed a disrupted lipid profile in the BALF of DPM-treated mice (Fig. 6g). Phosphatidylcholine (PC) 32:0, PC 34:1, PC 32:1, and PC 34:2, which account for approximately 60% of the total lipids pool in the BALF (Larsson et al. 2023), significantly accumulated in DPM-exposed mice (Fig. 6h). Most of the significantly altered ceramides (Cer) accumulated in the DPM group (Fig. 6h). Moreover, neutral lipids, particularly cholesterol, also showed significant accumulation (Fig. 6i). This represents a profound disruption in surfactant lipid homeostasis. These findings suggest that the capacity of AMs to clear pulmonary surfactant is impaired following DPM exposure. In vitro assays revealed that DPM-exposed MH-S cells exhibited significantly reduced BODIPY-cholesterol uptake (Fig. 6j). Since abnormal surfactant accumulation and AM dysfunction are hallmarks of pulmonary alveolar proteinosis (PAP) (Griese et al. 2019), these findings indicate that DPM exposure disrupts both surfactant protein and lipid homeostasis, thereby driving pathological changes characteristic of secondary PAP.

## Discussion

**Fig. 7:**
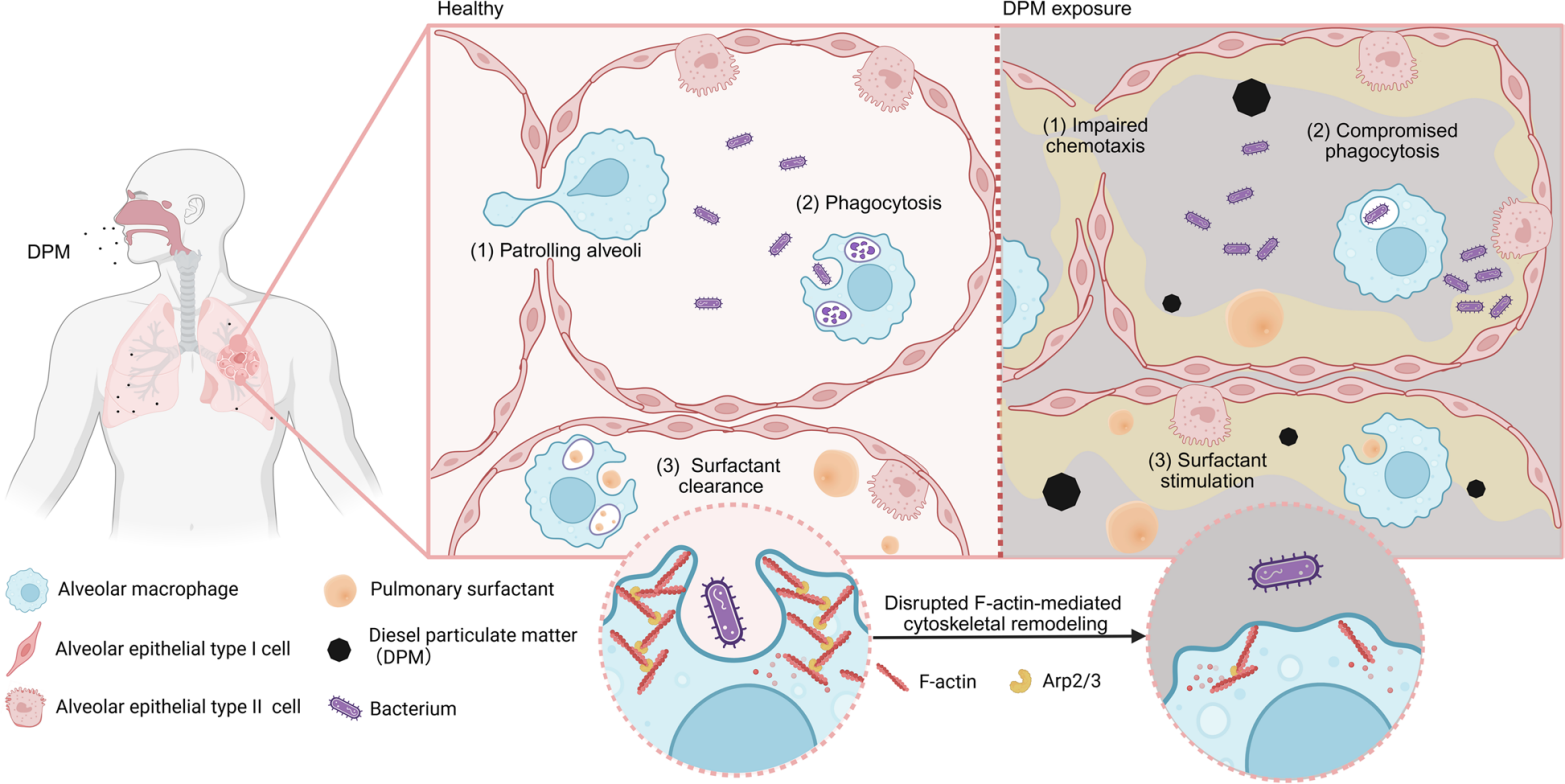
Schematic illustration of DPM-induced impairment of AMs function. TR-AMs, located in the alveolar lumen, act as key sentinels and effectors that maintain pulmonary homeostasis by patrolling the alveoli, phagocytosing pathogens, and clearing pulmonary surfactant. Inhaled DPM disrupts the functional integrity of TR-AM—specifically chemotactic migration and phagocytic activity—which depend on F-actin-mediated cytoskeletal remodeling. This functional impairment compromises immune surveillance and leads to surfactant accumulation in the alveoli, contributing to the development of secondary pulmonary alveolar proteinosis. Illustration created with *BioRender* (https://BioRender.com)

DPM is a major component of fine particulate matter (PM_2.5_), typically accounting for up to one-third of the PM_2.5_ in near-roadway or highly industrialized environments (Friedman 2020). Exposure to PM_2.5_ exerts deleterious effects across multiple organ systems. Among these, the pulmonary innate immune compartment is particularly susceptible (Losacco and Perillo 2018; Thangavel et al. 2022). Owing to its small aerodynamic diameter, PM_2.5_ can reach and accumulate deep within the alveolar spaces. This region serves as the first line of immune defense and the primary target for PM_2.5_-induced injury. In this study, we delineate the effects of DPM on AMs, highlighting their central role in maintaining pulmonary homeostasis. Our findings demonstrate that DPM exposure profoundly disrupts both immune and surfactant homeostasis within the alveolar niche (Fig. 7).

Maintaining pulmonary immune homeostasis is critically important yet inherently challenging. The lungs must continuously manage exposure to non-sterile air while minimizing excessive inflammation to preserve physiological integrity (Hartl et al. 2018). TR-AMs, comprising over 90% of alveolar immune cells, serve as the primary executors of immune surveillance (Cai et al. 2014; Gibbings et al. 2017). They constantly patrol the bronchi, bronchioles, and alveoli, efficiently phagocytosing inhaled particles, pathogens, debris, and apoptotic cells (Malainou et al. 2023; Wu et al. 2023). Indeed, nearly 90% of TR-AMs actively migrate between alveoli for routine patrolling (Neupane et al. 2020). This high degree of mobility is critical for rapidly intercepting and phagocytosing invading pathogens during early-stage infections (Neupane et al. 2020).

According to our results, DPM exposure significantly inhibits both the chemotaxis and phagocytosis of TR-AMs, likely by suppressing F-actin-mediated cytoskeletal remodeling. Given the central physiological role of TR-AMs, these findings provide new insights into how particulate exposure may disrupt pulmonary immune homeostasis. Under normal conditions, functional TR-AMs would swiftly clear such minor pathogens, without inducing the recruitment of circulating immune cells, thereby preventing an overt inflammatory response. However, impaired TR-AMs may fail to sense and clear low-level pathogens. This significantly increases the host’s susceptibility to infection. Furthermore, impaired phagocytic capacity might severely hinder the resolution of inflammation. During infection or chronic inflammatory conditions, DPM-impaired TR-AMs might fail to efficiently clear apoptotic neutrophils and damaged epithelial cells. This failure can lead to the release of noxious substances and exacerbate tissue damage. In fact, such defective phagocytosis is a common hallmark of various pulmonary diseases, including asthma (Fricker and Gibson 2017; Liang et al. 2014), cystic fibrosis (Alexis et al. 2006), and COPD (Berenson et al. 2013; Walsh 2008). Particularly in COPD patients, compromised AM engulfment of both pathogens and apoptotic cells drives persistent bacterial colonization and progressive inflammatory damage (Berenson et al. 2013; Donnelly and Barnes 2012; Walsh 2008). Future research should focus on exploring these potential pathological consequences of DPM exposure.

While previous studies linked PM_2.5_ exposure to macrophage phagocytic dysfunction, they largely relied on monocyte-derived macrophages (e.g., THP-1, RAW264.7) (Chen et al. 2020; Zhao et al. 2022), or uncharacterized lavage cells rather than specific TR-AMs (Zhang et al. 2022). Moreover, the impact of PM_2.5_ on the chemotactic motility of AMs remains largely unexplored. As a result, previous studies offer limited definitive insights into the effects of PM_2.5_ on alveolar immune surveillance. In the current study, by using both the MH-S cell line and mouse primary TR-AMs (CD45^high^SiglecF^+^), we provide direct evidence that DPM impairs both trans-migratory capacity and phagocytosis, underscoring the detrimental risks of environmental PM_2.5_ on alveolar immune homeostasis.

Although MH-S cells serve as a well-established in vitro model, they do not fully mirror all molecular features of primary TR-AMs. To ensure physiological relevance, we validated our key findings in vivo. DPM exposure induced a comparable and significant decrease in the phagocytic capacity of both MH-S cells and primary TR-AMs, suggesting a conserved core machinery for particle internalization. Regarding chemotaxis, while DPM dramatically inhibited MH-S mobility in vitro, we observed a similarly reduced population of highly phagocytic TR-AMs in vivo. However, these in vivo endpoint assays cannot fully distinguish whether this reduction arises from impaired motility or compromised phagocytic efficiency. Taken together, these findings demonstrate a functional consistency between MH-S cells and primary TR-AMs in response to DPM exposure. Nevertheless, the lack of direct evaluation of DPM’s impact on primary TR-AM chemotaxis remains a limitation of this study.

To further clarify the molecular mechanisms underlying the DPM-induced impairment of TR-AM phagocytic function, we focused on the dynamics of the actin cytoskeleton. Actin cytoskeletal remodeling provides the mechanical force required for both cellular motility and particle phagocytosis. In this study, pharmacological inhibition of actin polymerization using Cytochalasin D significantly suppressed MH-S phagocytosis. This process is mediated by the GTPase-NPFs-Arp2/3 signaling axis (Goley and Welch 2006; Rotty et al. 2013), a pathway whose vital role in macrophage phagocytosis has been widely documented. Li et al. reported that reduced F-actin polymerization in aged AMs contributes to diminished phagocytic capacity, and pharmacological inhibition of Rac1, N-WASP, or Arp2/3 significantly impairs bacterial clearance (Li et al. 2017). Similarly, Kong et al. demonstrated that inhibition of actin polymerization or the knockdown of actin markedly reduced LPS-induced Cdc42 and Rac activation and bacterial phagocytosis in macrophages (Kong and Ge 2008). In this study, DPM exposure significantly decreased the mRNA expression of key components of this pathway, including Rac1, Cdc42, Arp2, Arp3, and NPF family members WASP, N-WASP, and WAVE2 in MH-S cells. These findings implicate the suppression of the Rac1/Cdc42–WASP–Arp2/3 signaling cascade as a critical upstream driver of DPM-induced F-actin remodeling failure. However, the current evidence establishes a correlative mechanistic association rather than definitive causation. The lack of direct functional validation for this pathway remains a limitation. Future investigations employing pharmacological modulation or genetic manipulation would be valuable to further define the role of this signaling axis in DPM-induced AM dysfunction and the broader pathogenesis of lung injury.

Beyond immune regulation, TR-AMs critically contribute to pulmonary surfactant homeostasis by phagocytosing and catabolizing excessive surfactant within the alveoli. Impaired TR-AM phagocytosis leads to rapid surfactant accumulation. This was demonstrated by a sharp increase in lavage surfactant-phospholipid pools in AM-depleted rats (Forbes et al. 2007). Such clearance failure underlies the pathogenesis of PAP, a disease characterized by accumulated surfactant, impaired gas exchange, and increased susceptibility to infections (Trapnell et al. 2019). To date, research on PAP has predominantly focused on primary PAP, which arises from intrinsic defects in granulocyte–macrophage colony-stimulating factor (GM-CSF) signaling (Trapnell et al. 2009). However, secondary PAP remains underrecognized and frequently misdiagnosed, officially accounting for approximately 8%—10% of all PAP cases (Inoue et al. 2008; Ishii et al. 2011). Secondary PAP arises from external insults that reduce either the population or the functions of TR-AMs (Trapnell et al. 2019). Known causes include myelosuppression, inhalational syndromes, immunodeficiency disorders, and malignancies (Cordonnier et al. 1994; Seymour and Presneill 2002; Zhang et al. 2018). Notably, growing evidence implicates inhaled hazardous particles, including those from occupational exposure (Kumar and Cummings 2021; Xiao et al. 2015), airborne indium and its compounds (Kim et al. 2020; Li et al. 2020; Liu et al. 2022), and gallium oxide (Bomhard 2020), as potential contributors to secondary PAP.

In line with these observations, our results show that DPM inhibits cholesterol uptake by MH-S cells. Furthermore, in vivo DPM exposure led to a marked accumulation of surfactant proteins and lipids in BALF. This represents a common pathological feature associated with PAP (Griese et al. 2019). Notably, SP-D levels increased dramatically following DPM exposure, whereas SP-A levels exhibited a slight decline. Although both SP-A and SP-D participate in pulmonary innate immunity, their primary functions are highly divergent. SP-D primarily mediates innate host defense, while SP-A is essential for regulating surfactant lipid homeostasis and facilitating lipid clearance (Cañadas et al. 2020; Shamim et al. 2024). Therefore, the massive upregulation of SP-D may reflect an acute immune response to DPM-induced alveolar stress, while the concurrent reduction in SP-A could potentially compromise the AM-mediated clearance of alveolar lipids. Extensive surfactant accumulation forms a viscous granular substance that physically impedes ventilation, reducing lung compliance and causing hypoxemia. Furthermore, research indicates that elevated cholesterol impairs surface tension properties (Gunasekara et al. 2005), and its excessive uptake by AMs can trigger inflammasome activation and macrophage dysfunction (Vaso et al. 2026). Accumulated Cer may play a critical role in the pathogenesis of PAP by contributing to an apoptotic alveolar environment (Petrache et al. 2005). Collectively, these findings highlight a potential mechanistic link between particulate air pollution and the development of secondary PAP.

The critical role of TR-AM dysfunction in secondary PAP is increasingly supported by mechanistic studies. For instance, Jeon et al. demonstrated that exposure to In_2_O_3_ nanoparticles transformed AMs into foam cells, driving a secondary PAP-like phenotype characterized by extensive alveolar protein and phospholipid accumulation (Jeon et al. 2021). Kulle et al. reported that reduced AM mobility caused by exposure to e-cigarette vapor or CDC42 inhibition impaired surfactant clearance, resulting in protein accumulation within the alveoli (Kulle et al. 2024). Our research aligns with this paradigm. However, in this study, while the decreased cholesterol uptake in DPM-treated MH-S cells closely corresponds to the lipid accumulation in DPM-exposed mice, direct evidence linking this functional deficit to the observed pulmonary surfactant accumulation remains to be established. Future investigations aiming to restore TR-AM function, such as through pharmacological activation or genetic modulation of the Rac1/Cdc42 signaling axis, will be valuable to further define this causal link in DPM-induced lung injury. Furthermore, longitudinal studies are warranted to determine whether this DPM-induced surfactant dysregulation is transient or progresses to an irreversible phenotype. Such explorations will deepen our understanding of particulate-induced respiratory injury and secondary PAP pathogenesis.

In this study, the in vivo DPM exposure dose of 15 mg/kg via intratracheal instillation was guided by previously published murine models. These studies demonstrate that comparable doses up to 15—20 mg/kg effectively disrupt pulmonary homeostasis and trigger inflammatory responses (Jeong et al. 2021b; Wang et al. 2008; Yang et al. 2003a). To further understand the relevance of this dose in real-world scenarios, we estimated the equivalent burden by comparing the mass of particulate matter deposited per unit of alveolar surface area (Yanamala et al. 2013). Briefly, the cumulative murine dose of 30 mg/kg (two instillations of 15 mg/kg) corresponds to a deposition intensity of 10 mg/m^2^ in mice (0.06 m^2^ alveolar area). When scaled to a standard human lung (120 m^2^ alveolar area), this translates to an estimated total deposition of 1200 mg. Based on a standard human ventilation rate of 20 L/min and a deposition efficiency of 15%, this burden equates to roughly 6 days of occupational exposure (8 h/day) in underground mining environments with peak DPM concentrations of 144.17 mg/m^3^ (Yang et al. 2023b). However, this acute murine exposure protocol compresses days or months of cumulative exposure into concentrated instillations. This differs from actual human environmental or occupational settings, which typically involve long-term inhalation at much lower concentrations. Given this limitation, future studies employing long-term inhalation models are needed to better simulate actual exposure conditions and evaluate the health impacts of DPM.

Despite these limitations, our study demonstrates that TR-AM functional impairment is an important pathological factor affecting lung health under DPM exposure. Specifically, we found that exposure to DPM, a major constituent of ambient PM_2.5_, disrupts actin polymerization in AMs, thereby compromising their phagocytic capacity. This functional decline disrupts both the immune surveillance and surfactant homeostasis in the alveoli. Taken together, these findings indicate that respiratory infection susceptibility, unresolved inflammation, and secondary PAP may be significant pathological outcomes following PM_2.5_-induced lung injury. While the threat of PM_2.5_ to lung health is increasingly recognized, its specific contribution to these complex pathologies—particularly secondary PAP—remains to be fully defined. Advancing our understanding of how PM_2.5_ impairs TR-AM function and its subsequent role in respiratory homeostasis is of great value and significance. This represents a critical future perspective—one that we believe is not only urgent but also long overdue.

## Supplementary Information

Supplementary Material 1.

Supplementary Material 2.

## Data Availability

The RNA-seq data generated from MH-S cells are stored in the GSA database with the accession number CRA032505. Data will be made available on request.

## Abbreviations

AMs: Alveolar macrophages
BALF: Bronchoalveolar lavage fluid
CCL: C-C motif chemokine ligand
Cer: Ceramides
COPD: Chronic obstructive pulmonary disease
CXCL: C-X-C motif chemokine ligand
DEGs: Differentially expressed genes
DPM: Diesel particulate matter
*E. coli*: *Escherichia coli*
F-actin: Filamentous actin
GM-CSF: Granulocyte–macrophage colony-stimulating factor
IMs: Interstitial macrophages
LDH: Lactate dehydrogenase
MDMs: Monocyte-derived macrophages
MFI: Mean fluorescence intensity
NPFs: Nucleation-promoting factors
N-WASP: Neural WASP
PAP: Pulmonary alveolar proteinosis
PAS: Periodic acid-Schiff
PC: Phosphatidylcholine
PCA: Principal component analysis
PM_2.5_: Fine particulate matter
RFU: Relative fluorescence unit
RT-qPCR: Reverse transcription quantitative PCR
*S. aureus*: *Staphylococcus aureus*
SP-A: Surfactant protein A
SP-D: Surfactant protein D
TR-AMs: Tissue-resident alveolar macrophages
