# Supplementary material for: Phagocytotic impairment of tissue-resident alveolar macrophages by diesel particulates drives pulmonary surfactant accumulation
Source: Adv Biotechnol (Singap). 2026 May 19;4(2):21. doi: 10.1007/s44307-026-00113-y (PMC13187080; doi:10.1007/s44307-026-00113-y)
Supplement: Supplementary file 1 — Supplementary Material 1. [file 44307_2026_113_MOESM1_ESM.pdf]

Supplementary Figures

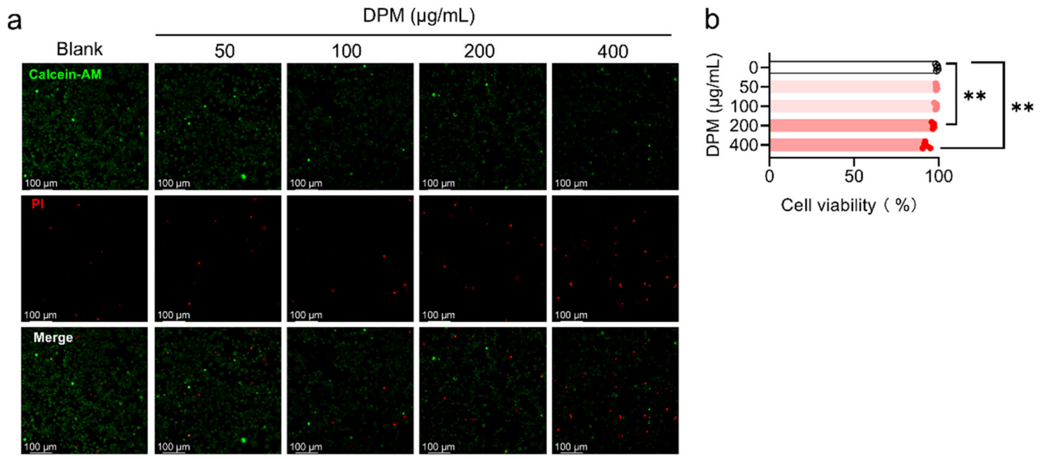

Fig. S1 Effect of different concentrations of DPM on MH-S cell viability. MH-S cells were treated with various doses of DPM for 24 hrs, (a) Representative immunofluorescence of Calcein-AM (green) and PI (red). (b) Statistics of cell viability (**$p < 0.01$, n=5)

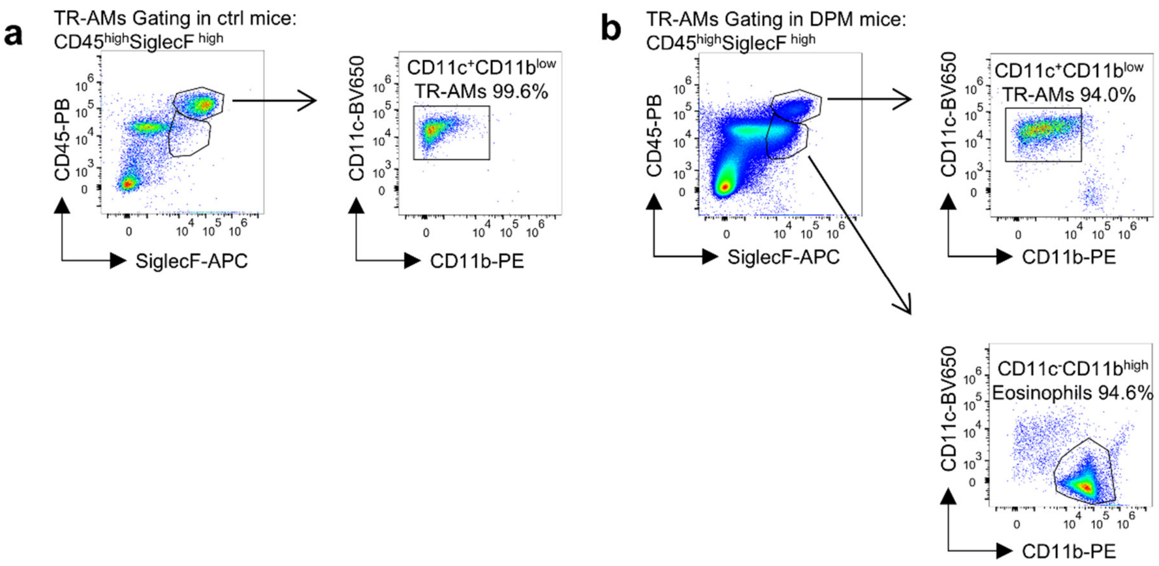

Fig. S2 Flow cytometry verification of TR-AMs purity. (a-b) Mouse primary TR-AMs were isolated from the BALF of vehicle (a) or DPM treated mice (b). After initial gating for single cells, TR-AMs were identified as $CD45^{high}SiglecF^{+}$. The CD11c and CD11b was evaluated to verify the purity of this population and $CD45^{int}SiglecF^{+}$ population

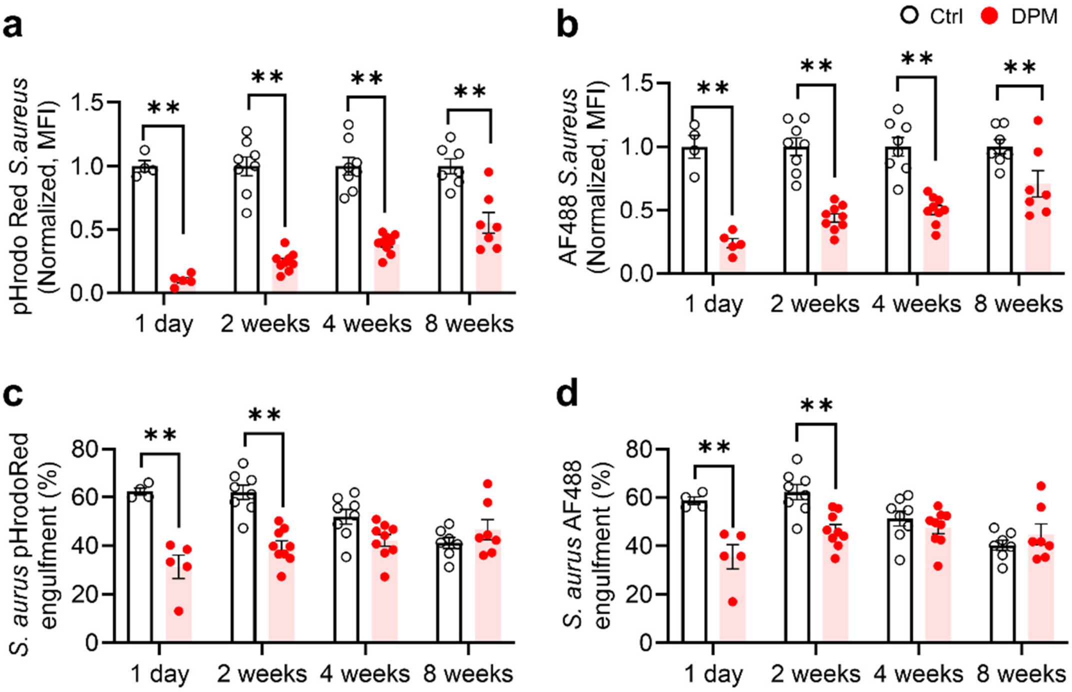

Fig. S3 Acute DPM exposure induces sustained impairment of alveolar macrophage phagocytosis. Mice were administered vehicle or DPM (15 mg/kg) via intratracheal instillation twice, at day 0 and 48 hrs. At the indicated time points after the final instillation (day1, 2 weeks, 4 weeks, and 8 weeks) post instillation, mice were intratracheally challenged with pHrodoRed-AF488-conjugated *S. aureus*. 3 hrs after the challenge, primary TR-AMs, identified as $CD45^{high}SiglecF^{+}$ singlets, were collected from BALF and gated for fluorescence quantification. (a–b) MFI of pHrodo Red and AF488 in TR-AMs was measured by flow cytometry as an index of phagocytic capacity. (c–d) The percentage of highly phagocytic TR-AMs was quantified (**p < 0.01, n = 4–9)

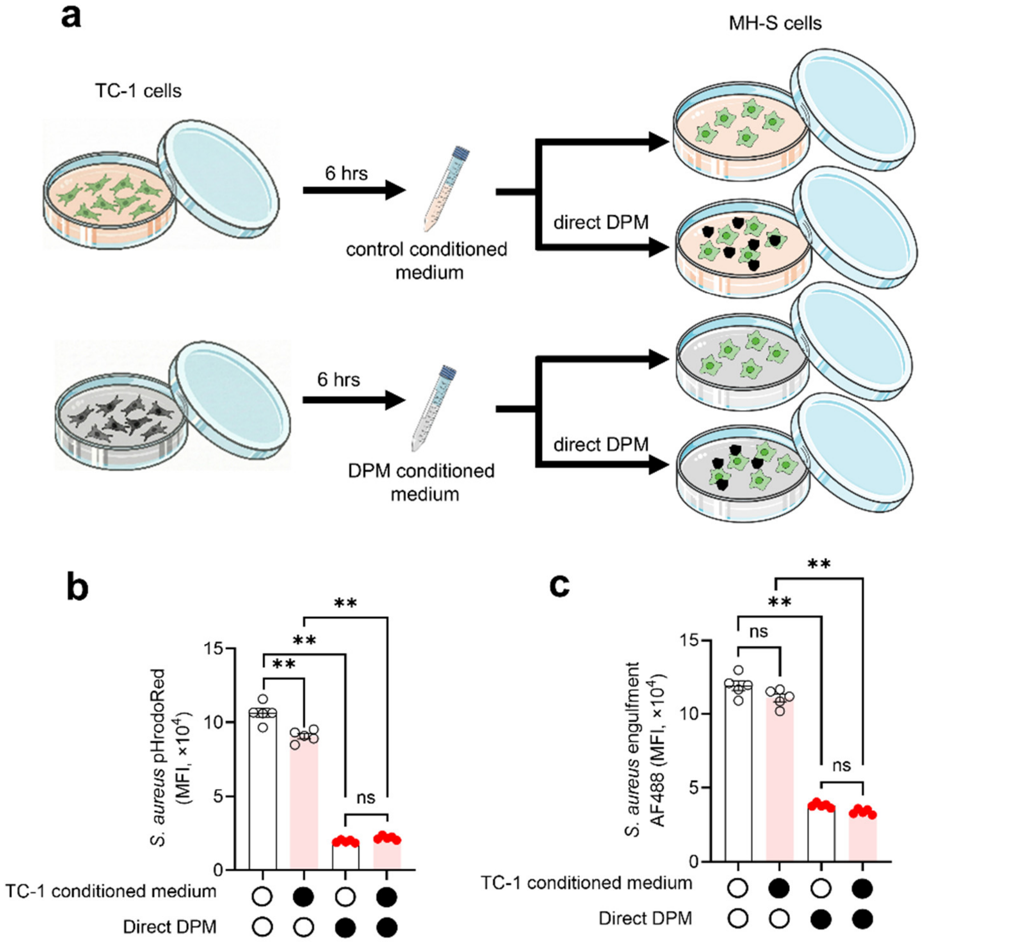

Fig. S4 The impairment of phagocytosis in MH-S is primarily driven by direct contact with DPM. (a) Schematic diagram of the experimental design. Murine lung epithelial cell line, TC-1 cells, were treated with vehicle or 200 µg/mL DPM for 6 hrs. Culture supernatants were collected, centrifuged, and filtered to make TC-1 ctrl or DPM conditioned medium. MH-S cells were incubated with the TC-1 conditioned medium with or without direct DPM exposure (200 µg/mL) for 24 hrs, followed by 1 hr co-incubation with AF488-pHrodo S. *aureus*. (b-c) Cellular phagocytosis was evaluated by flow cytometry, quantified by the MFI of pHrodoRed (b) and AF488 (c) (***p* < 0.01, ns: no significant, n=5)
