# Supplementary material for: Phagocytotic impairment of tissue-resident alveolar macrophages by diesel particulates drives pulmonary surfactant accumulation
Source: Adv Biotechnol (Singap). 2026 May 19;4(2):21. doi: 10.1007/s44307-026-00113-y (PMC13187080; doi:10.1007/s44307-026-00113-y)
Supplement: Supplementary file 2 — Supplementary Material 2. [file 44307_2026_113_MOESM2_ESM.docx]

Supplementary Table 1. Primer sequences for RT-qPCR

| Gene name | Forward (5’-3’) | Reverse (5’-3’) |
| --- | --- | --- |
| *Itgal* | GTCTCGGACATGCGATCAGAA | GGCGGGACGATTTTGTAACAT |
| *Itgam* | CCATGACCTTCCAAGAGAATGC | ACCGGCTTGTGCTGTAGTC |
| *Fpr1* | CACAATCCAAGTCCGTGAACG | CAGCTGTTGAAGAAAGCCAAGG |
| *Fpr2* | TTACAGCAGTTGTGGCTTCC | TAAACCAGACTGTGCCCAAA |
| *C5ar1* | CAGGCGGTGTAGAGGAGAAG | GAAGGAAGGAAGGAGGAGAGG |
| *C1qa* | TTCGGCAGAACCCAATGACG | TGGTATGGACTCTCCTGGTTG |
| *C1qb* | GGCAACCTGTGTGTGAATCTC | CTCTAGCTTCAAGACTACCCCA |
| *C1qc* | AGAAGCACCAGTCGGTATTCA | TGCGATGTGTAGTAGACGAAGTA |
| *RhoA* | AGCTTGTGGTAAGACATGCTTG | GTGTCCCATAAAGCCAACTCTAC |
| *Rac1* | GAGACGGAGCTGTTGGTAAAA | ATAGGCCCAGATTCACTGGTT |
| *Cdc42* | GCAGGGCAAGAGGATTATGA | CCCAACAAGCAAGAAAGGAG |
| *Was* | GCTTCGTGAAGGATAACCCTC | GGGTGGGAGTGAGATAAACCAG |
| *Wasl* | CTCGGCAAGAAATGTGTGACTA | ACTGCACTTCTTTGCCCACA |
| *Arp2* | CGACAACGGCACTGGGTTTG | ACTTGCCTCATCACCAACCAT |
| *Arp3* | TGGTGGTAGATTGAAGCCCA | AGCATTGACCCTCCAAACCA |
| *β-actin* | GCAGGAGTACGATGAGTCCG | ACGCAGCTCAGTAACAGTCC |
